# Can multi-modal radiomics using pretreatment ultrasound and tomosynthesis predict response to neoadjuvant systemic treatment in breast cancer?

**DOI:** 10.1007/s00330-023-10238-6

**Published:** 2023-09-14

**Authors:** Lie Cai, Chris Sidey-Gibbons, Juliane Nees, Fabian Riedel, Benedikt Schäfgen, Riku Togawa, Kristina Killinger, Joerg Heil, André Pfob, Michael Golatta

**Affiliations:** 1 Department of Obstetrics and Gynecology, Heidelberg University Hospital, Im Neuenheimer Feld 440, 69120 Heidelberg, Germany; 2 Department of Symptom Research, The University of Texas MD Anderson Cancer Center, Houston, TX USA; 3 MD Anderson Center for INSPiRED Cancer Care (Integrated Systems for Patient-Reported Data), The University of Texas MD Anderson Cancer Center, Houston, USA; 4 National Center for Tumor Diseases (NCT) and German Cancer Research Center (DKFZ), Heidelberg, Germany

**Keywords:** Breast cancer, Machine learning, Neoadjuvant systemic treatment, Treatment outcome

## Abstract

**Objectives:**

Response assessment to neoadjuvant systemic treatment (NAST) to guide individualized treatment in breast cancer is a clinical research priority. We aimed to develop an intelligent algorithm using multi-modal pretreatment ultrasound and tomosynthesis radiomics features in addition to clinical variables to predict pathologic complete response (pCR) prior to the initiation of therapy.

**Methods:**

We used retrospective data on patients who underwent ultrasound and tomosynthesis before starting NAST. We developed a support vector machine algorithm using pretreatment ultrasound and tomosynthesis radiomics features in addition to patient and tumor variables to predict pCR status (ypT0 and ypN0). Findings were compared to the histopathologic evaluation of the surgical specimen. The main outcome measures were area under the curve (AUC) and false-negative rate (FNR).

**Results:**

We included 720 patients, 504 in the development set and 216 in the validation set. Median age was 51.6 years and 33.6% (242 of 720) achieved pCR. The addition of radiomics features significantly improved the performance of the algorithm (AUC 0.72 to 0.81; *p* = 0.007). The FNR of the multi-modal radiomics and clinical algorithm was 6.7% (10 of 150 with missed residual cancer). Surface/volume ratio at tomosynthesis and peritumoral entropy characteristics at ultrasound were the most relevant radiomics. Hormonal receptors and HER-2 status were the most important clinical predictors.

**Conclusion:**

A multi-modal machine learning algorithm with pretreatment clinical, ultrasound, and tomosynthesis radiomics features may aid in predicting residual cancer after NAST. Pending prospective validation, this may facilitate individually tailored NAST regimens.

**Clinical relevance statement:**

Multi-modal radiomics using pretreatment ultrasound and tomosynthesis showed significant improvement in assessing response to NAST compared to an algorithm using clinical variables only. Further prospective validation of our findings seems warranted to enable individualized predictions of NAST outcomes.

**Key Points:**

• *We proposed a multi-modal machine learning algorithm with pretreatment clinical, ultrasound, and tomosynthesis radiomics features to predict response to neoadjuvant breast cancer treatment*.

• *Compared with the clinical algorithm, the AUC of this integrative algorithm is significantly higher*.

• *Used prior to the initiative of therapy, our algorithm can identify patients who will experience pathologic complete response following neoadjuvant therapy with a high negative predictive value*.

**Supplementary Information:**

The online version contains supplementary material available at 10.1007/s00330-023-10238-6.

## Introduction

Neoadjuvant systemic treatment (NAST) is the standard treatment for patients with early breast cancer because it allows response monitoring and tumor down-staging [1]. Patients who achieve a pathological complete response (pCR) have significantly better survival compared to non-pCR patients [2]. Understanding the likelihood of an individual will achieve pCR prior to the initiation of therapy could facilitate individually optimized NAST regimens.

The application of machine learning in medicine has developed rapidly in recent years [3]. Predicting tumor response to NAST in breast cancer has been explored in multiple radiomics studies [4–6]. Radiomics is a tool that can extract image features and present them numerically [7]. Currently, radiomics-based algorithms showed promising results in predicting breast tumor response but with certain limitations: (1) high performance is often seen for algorithms that use radiological examinations after/close to the completion of NAST, which limits the clinical application of the predictive algorithm; (2) most studies [8–10] investigated single-modality imaging radiomics which does not represent the integrative multi-modality imaging process in clinical routine (mainly ultrasound and mammography/tomosynthesis); (3) though MRI-based radiomics models showed satisfied results [9], MRI examinations are not routinely used for every patient due to contraindications and economic reasons [11]; (4) a lack of clearly reported, standardized imaging processing, which has a large effect on model performance and generalizability; (5) tomosynthesis has recently shown better performance compared to mammography in screening women with extremely dense breasts and at high risk of breast cancer [12], but the performance of tomosynthesis-based radiomics algorithms in response assessment to NAST remains unknown.

In this study, we aimed to develop and compare intelligent algorithms using multi-modal pretreatment ultrasound and tomosynthesis radiomics features in addition to clinical variables to predict pCR in breast cancer prior to the initiation of therapy.

## Methods

### Study design

This single-center and retrospective study was in accordance with the Declaration of Helsinki and was approved by the Ethics Committee of Heidelberg University Medical Faculty (S-092/2022).

In this study, we aimed to develop and compare intelligent algorithms using pretreatment ultrasound and tomosynthesis radiomics features in addition to clinical variables to predict response to NAST in breast cancer before the initiation of therapy. We considered three different types of input variables: clinical variables, ultrasound radiomics, and tomosynthesis radiomics. A full list and definition of clinical variables are detailed in Supplemental Table S2. Thus, we evaluated andcompared different models based on their input variables:

- Only clinical variables.
- Clinical variables and one-view ultrasound radiomics with peritumor information.
- Clinical variables and double-view ultrasound radiomics with peritumor information.
- Clinical variables and tomosynthesis radiomics.
- Clinical variables and tomosynthesis radiomics with peritumor information.
- Integrative, multi-modality model using clinical, ultrasound, and tomosynthesis radiomics including peritumor information.

### Patient selection

The inclusion criteria were as follows:

1. Patients with pathologically proven diagnosis of breast cancer.
2. Underwent neoadjuvant systemic treatment.
3. Underwent mammography tomosynthesis and ultrasound examination before neoadjuvant systemic treatment at Heidelberg University Hospital.
4. Without distant metastasis at the time of diagnosis.
5. Any tumor biology.

The exclusion criteria were as follows:

1. Combined with other tumor disease.
2. Aged <18 years.

Patients’ ultrasound and tomosynthesis images were acquired by experienced physicians specialized in breast diagnostics using Siemens machines (for ultrasound Siemens S2000 and S3000, for tomosynthesis Novation and Inspiration). The clearest double view of ultrasound images (view with largest diameter and 90° view) was documented, and one slice of tomosynthesis image in mediolateral oblique (MLO) view and mediolateral (ML) view with largest tumor was selected and documented. All images were stored in Digital Imaging and Communications in Medicine (DICOM) format. The corresponding clinical variables were documented from patients’ medical records (Table 1).

**Table 1:** Baseline clinical characteristics comparison between development set and validation set

Characteristics	Whole cohort (*n*=720)	Development set (*n*=504)	Validation set (*n*=216)	*p*
Age (SD)	51.6 (12.3)	51.5 (12.2)	51.8 (12.6)	0.778
Menopause status, no. (%)				
Premenopause	279 (38.8)	194 (38.5)	85 (39.5)	0.792
Perimenopause	105 (14.6)	76 (15.1)	29 (13.5)	0.751
Postmenopause	335 (46.6)	234 (46.4)	101 (47.0)	0.892
Missing	1	0	1	
Largest diameter on ultrasound before NAST (SD)	24.1 (12.7)	24.3 (12.8)	23.5 (12.4)	0.435
Largest diameter on tomosynthesis before NAST (SD)	28.7 (18.4)	28.7 (19.1)	28.5 (16.6)	0.883
Grading, no. (%)				
I	10 (1.4)	7 (1.5)	3 (1.4)	0.992
II	29 (42.5)	206 (42.7)	87 (41.8)	0.574
III	387 (56.1)	269 (55.8)	118 (56.7)	0.820
Missing	30	22	8	
Tumor type, no. (%)				0.143
Invasive	714 (99.3)	501 (99.6)	213 (98.6)	
In situ	5 (0.7)	2 (0.4)	3 (1.4)	
Missing	1	1	0	
Tumor histology, no. (%)				
Invasive ductal carcinoma	637 (95.6)	448 (96.1)	189 (94.5)	0.343
Invasive lobular carcinoma	28 (4.2)	18 (3.9)	10 (5.0)	0.503
Invasive medullary carcinoma	1 (0.2)	0	1 (0.5)	-
Missing	54	38	16	
Estrogen receptor, no. (%)				0.046*
Positive	412 (57.7)	300 (60.1)	112 (52.1)	
Negative	302 (42.3)	199 (39.9)	103 (47.9)	
Missing	6	5	1	
Progesterone receptor, no. (%)				0.546
Positive	380 (53.4)	270 (54.1)	110 (51.6)	
Negative	332 (46.6)	229 (45.9)	103 (48.4)	
Missing	8	5	3	
HER-2, no. (%)				0.665
Positive	296 (41.6)	204 (41.0)	92 (42.8)	
Negative	416 (58.4)	293 (59.0)	123 (57.2)	
Missing	8	7	1	
Breast density, no. (%)				
Fatty	36 (6.0)	29 (1.5)	7 (3.9)	0.154
Scattered fibroglandular tissues	298 (49.7)	207 (42.7)	91 (50.6)	0.776
Heterogeneous dense	209 (34.8)	138 (55.8)	71 (39.4)	0.121
Extremely dense	57 (9.5)	46 (11.0)	11 (6.1)	0.066
Missing	120	84	36	
cT, no. (%)				
T1	230 (32.6)	158 (31.9)	72 (34.4)	0.502
T2	353 (50.1)	250 (50.4)	103 (49.3)	0.786
T3	75 (10.6)	52 (10.5)	23 (11.0)	0.838
T4	47 (6.7)	36 (7.3)	11 (5.3)	0.332
Tx	15	8	6	
cN, no. (%)				
cN0	410 (58.6)	279 (56.9)	131 (62.4)	0.180
cN1	206 (29.4)	147 (30.0)	59 (28.1)	0.612
cN2	45 (6.4)	32 (6.5)	13 (6.2)	0.866
cN3	39 (5.6)	32 (6.5)	7 (3.3)	0.091
cNx	20	14	6	0.761
Ki-67 (SD)	51.0 (25.4)	52.3 (25.7)	48.1 (24.5)	0.055
Karnofsky Index (SD)	97.5 (6.6)	97.5 (6.9)	97.4 (5.8)	0.880
Outcome, no. (%)				0.256
pCR	242 (33.6)	176 (34.9)	66 (30.6)	
Non-pCR	478 (66.4)	328 (65.1)	150 (69.4)	

Abbreviations: *SD*, standard deviation; *NAST*, neoadjuvant systemic treatment; *HER-2*, human epidermal growth factor receptor 2; *pCR*, pathologic complete response

### Image processing

1. Histogram matching

We used histogram matching to maintain consistency of images acquired by different types of machines and settings [13], and one normal ultrasound image and one slice of normal tomosynthesis image were selected for histogram matching.

2) Segmentation

**Fig. 1:**
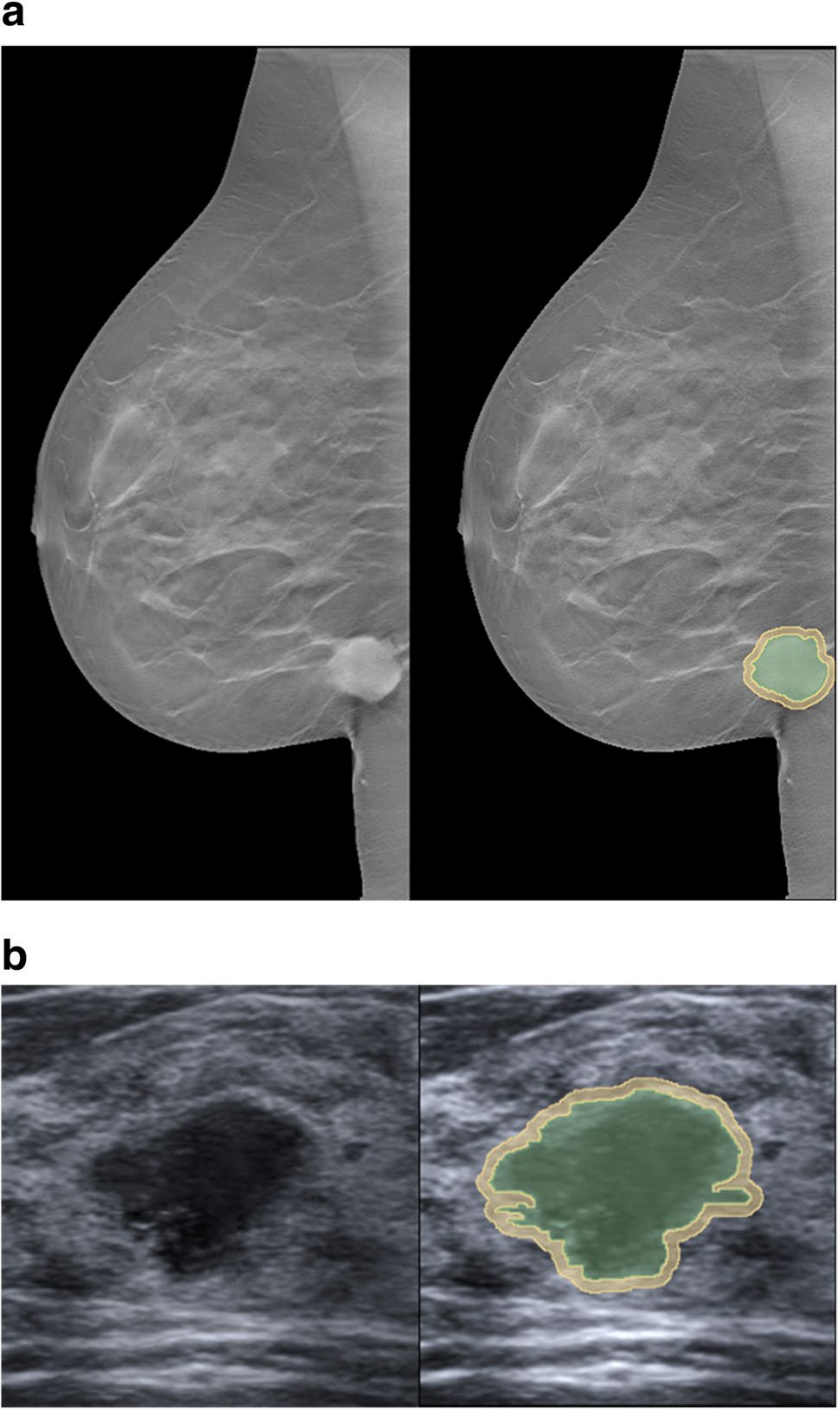
Examples of segmentation in tomosynthesis and ultrasound images. **a** Tomosynthesis. **b** Ultrasound. Tumor segmentations (green) were delineated semi-automatically; peritumor segmentations (yellow) were generated by “Hollow” effect

We used the open-source software 3D slicer (4.13.0-2022-04-01) for segmentation, the outline of tumors was delineated semi-automatically, and the 3-mm peritumor spaces were generated by using the “Hollow” effect in 3D slicer [14]. Figure 1 shows examples of segmentation in an ultrasound image and a tomosynthesis image.

3) Re-segmentation, discretization, and feature extraction

Re-segmentation and discretization were done at the same time when doing feature extraction. Re-segmentation was performed to remove pixels from the segmented region that fall outside of the specified range of gray levels to reduce errors caused by manual delineation [15]. Discretization is conceptually equivalent to the creation of a histogram to make feature calculation tractable [16]. They were shown as parameters of feature extraction on the practical level. We used the most common parameter ***μ±3σ*** for re-segmentation [15, 17] (***μ*** stands for the mean value of gray levels and ***σ*** stands for the standard deviation). The optimal number of bin widths for image discretization is still unclear [18]; we set the bin width as 10 for discretization. We used the open-source software Pyradiomics for feature extraction [19].

4) Feature selection

We used Pearson’s correlation coefficient matrix (PCCM) and recursive feature elimination (RFE) embedded within the 10-fold of cross-validation on the development set for feature selection. First, PCCM was applied to identify multicollinearity between features; only one feature was preserved of any pair with a correlation coefficient of more than 0.85 or less than −0.85 [20]. Second, RFE was applied to further reduce the number of radiomics features on the development set [21] [22].

### Outcome and definitions

Pathological evaluation of the surgical specimen served as gold standard for the definition of pCR. We assumed pCR if no residual invasive or in situ tumor cells were found in the breast and axillary lymph nodes (ypT0 and ypN0). Details are shown in Table 1.

### Model construction and evaluation

For the algorithm development and reporting, we considered guidelines on machine learning in medicine [23], diagnostic tests [24], and multivariable prediction models [25]. A checklist informed by recent guidelines on machine learning in medicine is provided in the Data Supplement.

We chose a supporting vector machine (SVM) algorithm for model construction due to its known characteristic of considering non-linear inter-feature relationships [26]. We randomly split the whole cohort into a development set (504 of 720, 70.0%) and a validation set (216 of 720, 30.0%).

**Fig. 2:**
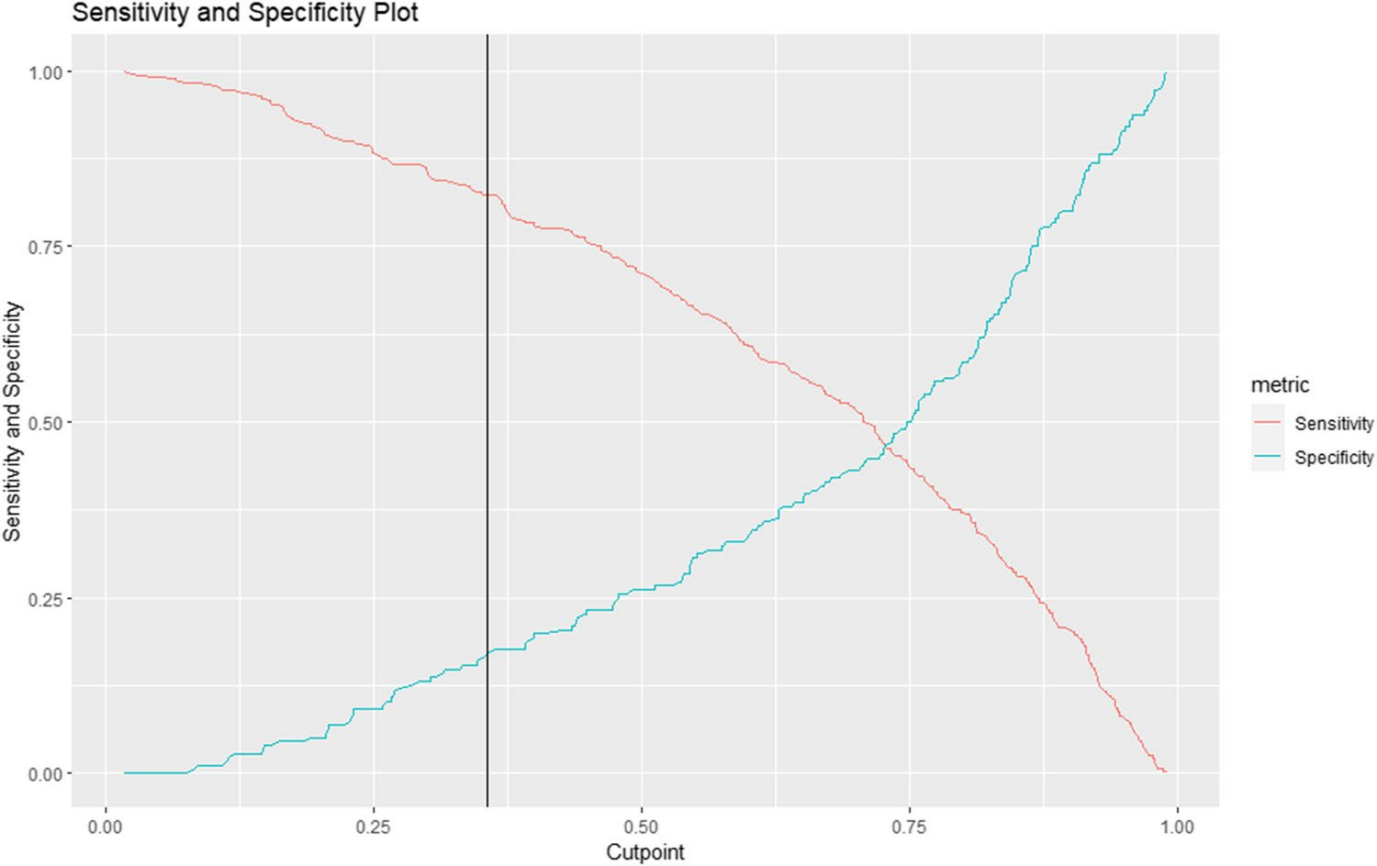
Cutoff chosen on the integrative multi-modal model in development set

Ten-fold cross-validation was used for the algorithm training and internal testing on the development dataset. A hypergrid-search was performed to select the optimal hyperparameters. False-negative rate (FNR) was considered the main measurement of model performance. The risk threshold for the binary outcome prediction was chosen at 90% sensitivity in the development set by maximizing the metric with 1000 times bootstrap replicates. The final integrative multi-modal model with an optimized threshold was then validated using the validation set. Figure 2 illustrates the cutoff chosen for the final integrative multi-modal model in the development set.

We calculated additional diagnostic metrics like area under the receiving operator curve (AUC), accuracy, specificity, sensitivity, false-positive rate (FPR), positive predictive value (PPV), and negative predictive value (NPV). We used the “DALEX” package in R to calculate the agnostic variable importance measure computed via permutation (e.g., computing the loss function for the full model and then computing randomized response variables’ loss function). We used decision curve analysis (DCA) to better illustrate the benefits of clinical application of the models [27]. Python (Version 3.9.7) and R (Version 4.2.1) were used for all analyses.

### Statistical analysis

We performed a descriptive analysis to illustrate the distribution of the baseline characteristics of the development set and validation sets. We used the chi-square test for categorical data, and the *t*-test for continuous data to compare differences in baseline characteristics between the development and validation set. We calculated area under the receiver operating characteristic curve and accompanying 95% CIs for the algorithms using 2000 bootstrap replicates stratified for the outcome variable (non-pCR, ypT+, and/or ypN+). The Venkatraman method tests were used to compare models’ performance [28]. A proportion test was used to compare the model’s diagnostic performance [29]. Calibration plots (observed vs. predicted probabilities) and Spiegelhalter’s *Z* statistics were used to evaluate model calibration [30, 31].

*p* values < 0.05 were considered significant.

## Results

### Patient flow

**Fig. 3:**
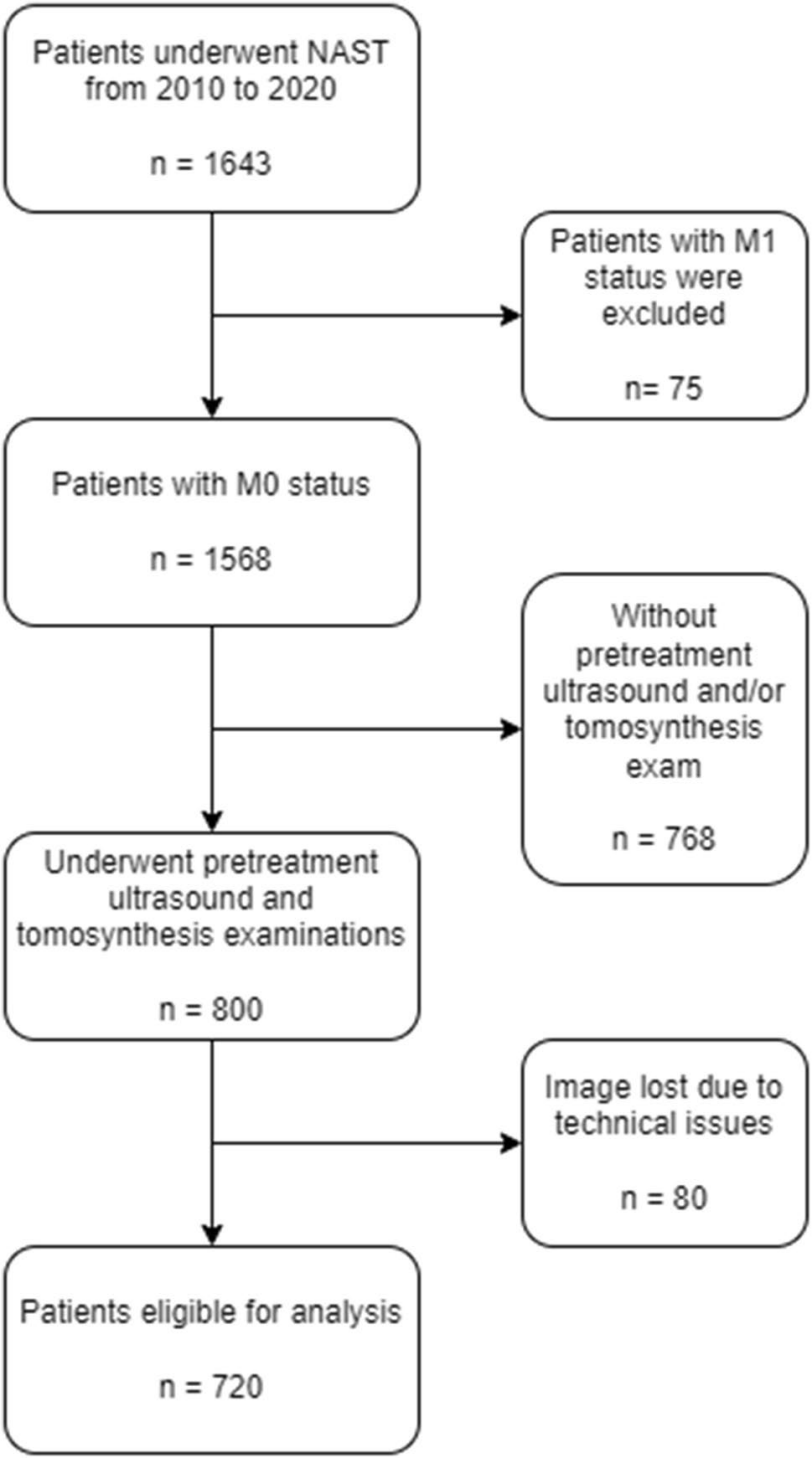
Diagram of patients selection

Of 1643 patients who underwent neoadjuvant systemic treatment from 2010 to 2020 at Heidelberg University Hospital, 75 were excluded because of distant metastasis, 768 were excluded because they did not undergo pretreatment ultrasound and/or tomosynthesis examinations at our institution, and 80 were lost due to technical issues (not transferable into image analysis software or double-view ultrasound images saved side-by-side within one image instead of two separate images). The remaining 720 patients were analyzed in this study (Fig. 3).

### Baseline characteristics

Of 720 patients, 33.6% (242 of 720) achieved pCR. Comparing the development and validation sets, more patients in the development had ER-positive tumors (60.1% vs. 52.1%, *p* = 0.046). Details regarding baseline clinical characteristics are shown in Table 1. pCR rates according to breast cancer subtype are displayed in Table 2. Her-2 over-expression subtype achieved the highest pCR rate in the whole cohort (49 of 79, 62.0%) and the development set (35 of 51, 68.6%).

**Table 2:** pCR rate according to breast cancer subtypes

		TNBC	HER-2	Luminal	All subtypes
Whole cohort (*n* = 720)	pCR	78	49	115	242
	Non-pCR	103	30	345	478
	pCR rate	43.10%	62.00%	31.90%	33.60%
Development set (*n* = 504)	pCR	53	35	88	176
	Non-pCR	71	16	241	328
	pCR rate	42.70%	68.60%	26.70%	34.90%
Validation set (*n* = 216)	pCR	25	14	27	66
	Non-pCR	32	14	104	150
	pCR rate	43.90%	50.00%	20.60%	30.70%

Abbreviations: *TNBC*, triple-negative breast cancer; *HER-2*, human epidermal growth factor receptor 2 over expression subtype; *pCR*, pathological complete response

### Feature selection

Per segmentation, 130 features were extracted, resulting in a total of 780 features for one patient with double-view ultrasound and tomosynthesis, with tumor as well as peri-tumor segmentation. After removing non-numeric features by applying PCCM, 22 ultrasound radiomics features and 33 tomosynthesis radiomics features were preserved. Finally, 23 features were selected by RFE, detailed in Table S3. The final model features are provided in Table S4.

### Model performance

**Fig. 4:**
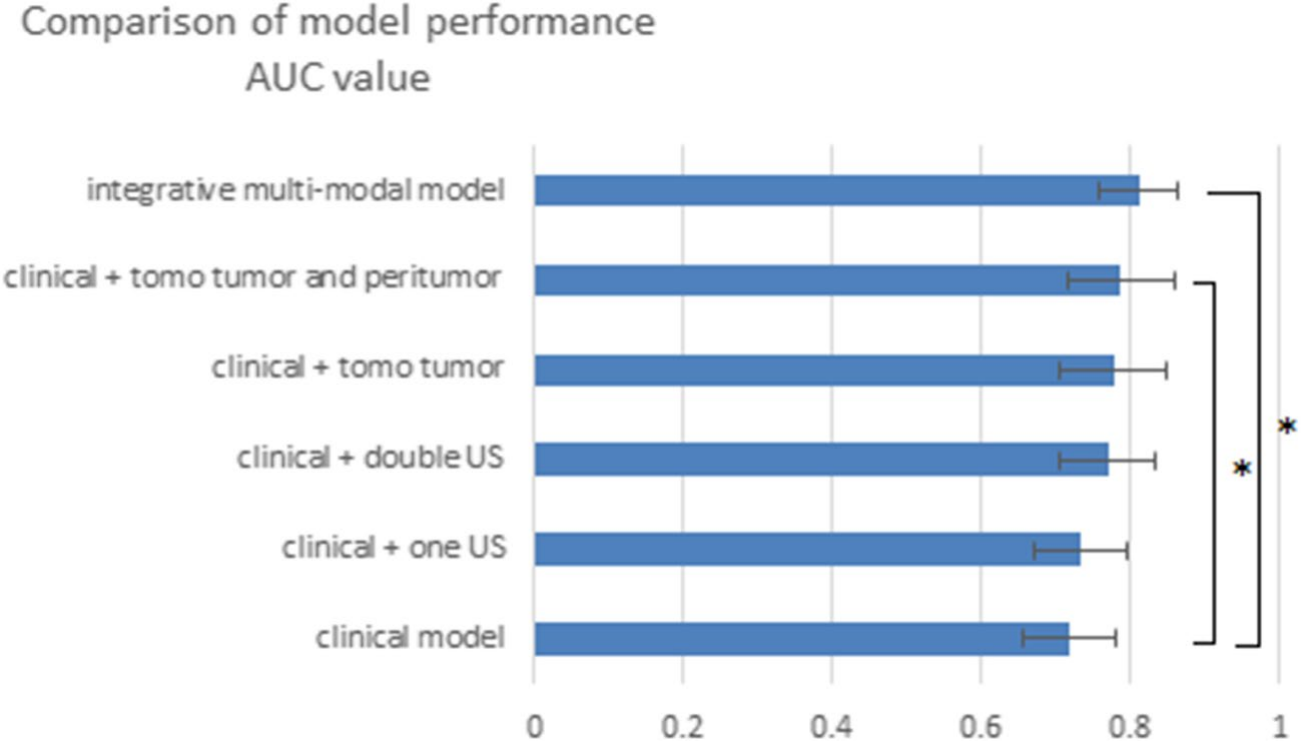
Comparison of model performance. * stands for statistical significant difference between models. Abbreviation: AUC, area under the curve; US, ultrasound; tomo, tomosynthesis

Figure 4 shows the comparison between the different SVM models: the clinical model, one-view ultrasound model, two-view ultrasound model, tomosynthesis tumor radiomics model, tomosynthesis tumor plus peritumor radiomics model, and the integrative model with multi-modal clinical, ultrasound, and tomosynthesis radiomics features. The multi-modal model and the model with tomosynthesis tumor plus peritumor radiomics features had significantly higher performance in predicting tumor response to NAST compared to the clinical model (AUC 0.81, 95% CI 0.75–0.87 and AUC 0.79, 95% CI 0.72–0.85, respectively, vs. 0.72, 95% CI: 0.65–0.78; *p* = 0.007 and *p* = 0.03). The rest of the models’ AUC values were improved without statistical significance (Table S4). When ypT0/is, ypN0 was used as endpoint definition, and the integrative multi-modal model performance was AUC 0.78 (95% CI 0.71–0.85; see Table S6).

With an eye to reliably excluding residual cancer after NAST, the multi-modal model revealed a significantly lower FNR of 6.7% (10 of 150 patients with missed residual cancer), compared to the clinical model (14.0%, 21 of 150, *p* = 0.016). Table 3 shows the diagnostic performance metrics of the clinical model and multi-modal model.

**Table 3:** Diagnostic performance of clinical model and the integrative multi-modal model

	Clinical model	Integrative multi-modal model	*p*
SVM–AUROC (95% CI)	0.72 (0.65–0.78)	0.81 (0.75–0.87)	0.007*
Accuracy	69.9% (151 of 216)	76.4% (165 of 216)	0.079
FNR	14.0% (21 of 150)	6.7% (10 of 150)	0.016*
FPR	66.7% (44 of 66)	62.1% (41 of 66)	0.500
PPV	74.6% (129 of 173)	77.3% (140 of 181)	0.687
NPV	51.2% (22 of 43)	71.4% (25 of 35)	0.056
Sensitivity	86.0% (129 of 150)	93.3% (140 of 150)	0.029*
Specificity	33.3% (22 of 66)	37.9% (25 of 66)	0.358

* stands for statistical significance between models by Venkatraman tests

Abbreviations: *SVM*, supporting vector machines; *CI*, confidence interval; *FNR*, false-negative rate; *FPR*, false-positive rate; *PPV*, positive predictive value; *NPV*, negative predictive value

Table 4 shows the matrix of the clinical model and multi-modal model as well as AUC values by tumor biologic subtype. The luminal subtype achieved the highest AUC of 0.83 (95% CI: 0.75–0.91) and the TNBC subtype achieved the lowest AUC (0.71, 95% CI: 0.57–0.83).

**Table 4:** Matrix of clinical model and the integrative multi-modal model in different subtypes

			Pathologically confirmed		AUC (95% CI)
			Residual cancer (*n* = 150)	No residual cancer (*n* = 66)	
Clinical model prediction on validation set	*n* = 216	Residual cancer	129	44	0.72 (0.65–0.78)
		No residual cancer	21	22	
Integrative model prediction	TNBC	Residual cancer	29	7	0.71 (0.57–0.83)
	*n* = 57	No residual cancer	3	18	
	HER-2	Residual cancer	11	10	0.77 (0.57–0.92)
	*n* = 28	No residual cancer	3	4	
	Luminal	Residual cancer	100	24	0.83 (0.75–0.91)
	*n* = 131	No residual cancer	4	3	
	Invasive lobular carcinoma	Residual cancer	7	1	0.71 (0.14–1.00)
	*n* = 10	No residual cancer	0	2	
	Invasive ductal carcinoma	Residual cancer	119	39	0.80 (0.73–0.86)
	*n* = 189	No residual cancer	10	21	
	whole validation set	Residual cancer	140	41	0.81 (0.75–0.87)
	*n* = 216	No residual cancer	10	25	

Abbreviations: *TNBC*, triple-negative breast cancer; *HER-2*, human epidermal growth factor receptor 2 over expression subtype; *AUC*, area under the curve; *CI*, confidence interval

### Insights into model predictions

Table 5 illustrates the clinical univariable and multivariable logistic regression results of non-pCR versus pCR. Upon performing multivariable logistic regression, Ki-67 (odds ratio [OR] 0.99; 95% CI, 0.98 to 1.00, *p* = 0.003), perimenopause status (OR 0.54; 95% CI, 0.31 to 0.95, *p* = 0.032), positive estrogen receptor (ER) status (OR 1.82, 95% CI, 1.15 to 2.89, *p* = 0.011), positive progesterone receptor (PR) status (OR 2.14, 95% CI, 1.35 to 3.40, *p* = 0.001), and positive HER-2 status (OR 0.32, 95% CI, 0.22 to 0.47, *p* < 0.001) were significantly associated with non-pCR after NAST.

**Table 5:** Association of clinical model variables with non-pCR in univariable and multivariable analysis

Characteristics	Univariable logistic regression		Multivariable logistic regression	
	OR (95% CI)	*p*	OR (95% CI)	*p*
Age	1.01 (0.99, 1.02)	0.300	1.01 (0.99, 1.02)	0.348
Ki-67	0.98 (0.97, 0.99)	<0.001	0.99 (0.98, 1.00)	0.003*
Karnofsky Index	0.98 (0.96, 1.01)	0.258	0.99 (0.96, 1.02)	0.507
Largest diameter on ultrasound before NAST	1.01 (0.99, 1.02)	0.397	1.01 (0.98, 1.02)	0.851
Largest diameter on tomo-synthesis before NAST	1.00 (0.99, 1.01)	0.418	1.00 (0.99, 1.01)	0.937
Menopause status				
Premenopause	Reference		Reference	
Perimenopause	0.78 (0.49, 1,24)	0.296	0.54 (0.31, 0.95)	0.032*
Postmenopause	1.06 (0.75, 1.48)	0.754	0.69 (0.37, 1.28)	0.236
cT				
T1	Reference		Reference	
T2	1.18 (0.84, 1.66)	0.351	1.18 (0.77, 1.81)	0.435
T3	2.07 (1.13, 3.77)	0.018	1.87 (0.83, 4.23)	0.133
T4	1.58 (1.03, 5.53)	0.192	1.93 (0.77, 4.80)	0.159
cN				
cN0	Reference		Reference	
cN1	0.95 (0.67, 1.35)	0.778	0.99 (0.66, 1.50)	0.986
cN2	1.08 (0.56, 2.06)	0.821	1.04 (0.49, 2.19)	0.919
cN3	2.38 (1.03, 5.53)	0.043	1.56 (0.60, 4.01)	0.359
ER				
Negative	Reference		Reference	
Positive	3.14 (2.32, 4.40)	<0.001	1.82 (1.15, 2.89)	0.011*
PR				
Negative	Reference		Reference	
Positive	3.26 (2.36, 4.50)	<0.001	2.14 (1.35, 3.40)	0.001*
HER-2				
Negative	Reference		Reference	
Positive	0.54 (0.40, 0.74)	<0.001	0.32 (0.22, 0.47)	<0.001*
Grading				
I	Reference		Reference	
II	0.42 (0.05, 3.37)	0.414	0.64 (0.07, 5.49)	0.680
III	0.14 (0.02, 1.15)	0.067	0.35 (0.04. 3.04)	0.342
Breast density				
Fatty	Reference		Reference	
Scattered dense	1.24 (0.54, 2.85)	0.805	1.36 (0.52, 3.57)	0.897
Heterogeneous dense	1.42 (0.69, 2.92)	0.336	1.45 (0.65, 3.25)	0.367
Extremely dense	1.09 (0.54, 2.19)	0.607	1.05 (0.49, 2.27)	0.530
Tumor type				
In situ	Reference		Reference	
Invasive	0.49 (0.05, 4.42)	0.527	0.32 (0.03, 3.54)	0.354

Abbreviations: *NAST*, neoadjuvant chemotherapy; *OR*, odds ratio; *CI*, confidence interval; *ER*, estrogen receptor; *PR*, progestogen receptor; *HER-2*, human epidermal growth factor receptor 2

**Fig. 5:**
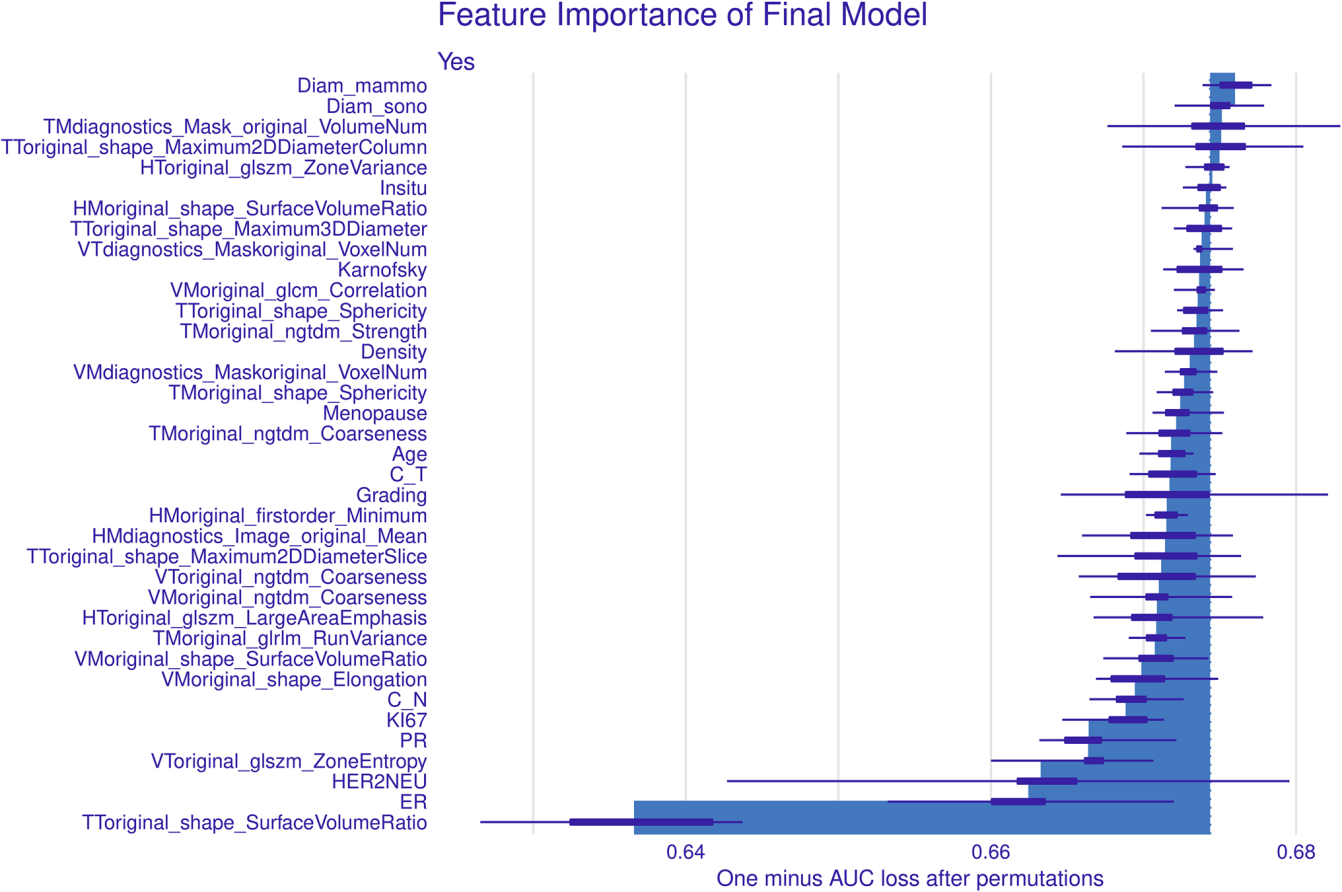
Insights into variable importance of the integrative multi-modal model. Abbreviations: Diam_mammo, largest diameter on tomosynthesis before NAST; Diam_sono, largest diameter on ultrasound before NAST; TT, tomosynthesis tumor features; TM, tomosynthesis peritumor features; HT, first view of ultrasound tumor features; HM, first view of ultrasound peritumor features; VT, second view of ultrasound tumor features; VM, second view of ultrasound peritumor features

Figure 5 illustrates insights into the variable importance for the predictions made by the multi-modal SVM model. The five most important variables were tomosynthesis tumor original shape surface volume ratio, ER status, HER-2 status, ultrasound tumor original gray level size zone matrix (GLSZM) zone entropy, and PR status.

**Fig. 6:**
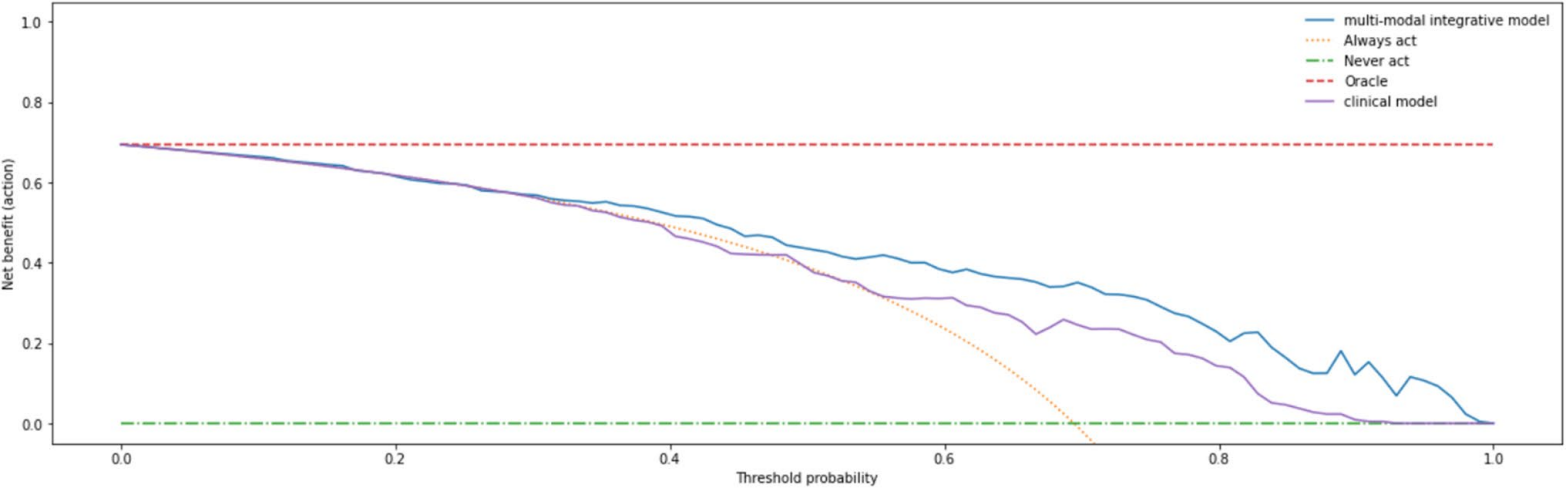
Decision curve analysis comparing the integrative multi-modal model and the clinical model

Figure 6 shows the decision curve analysis of the integrative multi-modal model and the clinical model. Net benefits of the two models and the default approaches of treating all (always act) patients or treating none (never act) patients are shown. From 0.29 to 1.0 threshold probabilities, the integrative multi-modal model has the highest net benefit.

### Model calibration

Figure S2 illustrates the calibration plot of the multi-modal SVM model; Spiegelhalter’s *z* indicates a well calibrated model (*z* = 0.2301, *p* = 0.409).

## Discussion

We developed and compared intelligent algorithms using multi-modal pretreatment ultrasound and tomosynthesis radiomics features in addition to clinical variables to predict response to NAST in breast cancer prior to treatment initiation. The integrative, multi-modal algorithm showed significant improvement in assessing response to NAST compared to an algorithm using clinical variables only (AUC 0.81, 95% CI 0.75–0.87 vs. AUC 0.72, 95% CI 0.65–0.78, *p* = 0.007) with a FNR of 6.7% (10 of 150 patients with missed residual cancer in the surgical specimen, ypT+ or ypN+). To our knowledge, this is the first study to use multi-modal radiomics features from different examinations to create predictions prior to treatment. Our study strictly follows the Image Biomarker Standardization Initiative (ISBI) guideline [15], and presents transparent parameters of image processing (i.e., histogram matching, image re-segmentation, and discretization).

Individualized treatment for breast cancer patients undergoing NAST has been a research priority over the past decade. Although up to 60% of patients achieve pCR (depending on tumor size and biology) [32], every patient currently has to undergo surgery due to the lack of tools to reliably exclude residual cancer prior to surgery. A recent single-center study reported the first oncologic outcomes for the omission of breast surgery using a vacuum assisted biopsy (VAB) performed after NAST in patients with strict inclusion criteria (cT1-2, cN0-1, triple-negative or HER-2 positive, residual lesion < 2 cm on imaging after NAST): There was no ipsilateral recurrence at a follow-up of 26.4 months [33]. However, the use of VAB previously showed high FNR in a multi-center setting [34]. Recently, a multicenter, intelligent VAB algorithm showed a FNR of 0.0–5.2% [35]. Our present study showed comparable results (FNR: 6.7%, 10 of 150) without the use of an additional biopsy procedure and with only pretreatment information.

Expanding on this clinical background, potential new pathways for the addressed patients following NAST are imaginable: The real-world scenario currently directs all patients to surgery following NAST and accepts high rates of overtreatment (surgery) for histological negative patients, but avoids undertreatment using the integrative multi-modal model. All test-positive patients might be directed to surgery, resulting in overtreatment of false-positive patients (41/181; 22.6%; false positives), which is however still lower compared to the current practice (100% undergo surgery). All test-negative patients might be directed to extended non-invasive biopsy. Undertreatment of false-negative patients (10/35; 28.5%) must be avoided and might be prevented by extended imaging-guided vacuum-assisted biopsy of the tumor bed or radiation therapy and omitting surgery. Patients with positive biopsy results would need to be directed to surgery. Finally, 11.6% (25/216) would benefit from this de-escalating concept; this proportion is in line with recent paradigm shifts in locoregional breast cancer management [36]. It should be noted, however, that the NPV of 71.6% means that 28.4% of patients who have been told a negative (tumor-free) test result might skip surgery although there is actually residual cancer left. Notably, past surgical de-escalation strategies in breast cancer were based on the FNR, as the FNR is independent from the prevalence in the respective population.

Many studies have tried to build radiomics models to predict tumor response to NAST, but their performances and qualities vary [37]. In terms of performance, features extracted from multiple times of examinations performed better, with AUCs ranging from 0.86 [38] to 0.94 [5], but require patients to undergo several examinations (i.e., pretreatment, early treatment—after completion of two [38] and/or four cycles [8] of NAST—and post-treatment). This requires a high degree of patient compliance and consumes a great deal of effort by physicians in clinical application. In terms of quality, some studies extracted features from a single timeframe of examination but not reported a specific time [39–41]. Other studies developed models only with pretreatment radiomics features, with performances ranging from 0.79 [42] to 0.92 [43]. However, sample sizes remained limited [25] (development set up to 362 patients [43]).

The peritumor space is considered to be highly related to the tumor microenvironment and plays an important role in the process of tumor angiogenesis and proliferation [44]. Radiomics studies based on MRI [9], ultrasound [10], and mammography [39] demonstrated that peritumor space can provide complementary information for predicting tumor response. But the optimal width of peritumor space remains controversial, with some studies suggesting that wider peritumor space (10 mm) performed worse than narrower space (5 mm) [39]. Few studies investigated the efficacy of peritumor space on tomosynthesis. In our study, we extracted features from 3 mm peritumor space, and the performance of the tomosynthesis tumor plus peritumor model improved but without statistical significance compared to the tomosynthesis tumor-only model.

There is an ongoing discussion about whether radiomics or deep learning analyses should be preferred for the analysis of medical images. Deep learning analyses often show higher performance and require less human work during the image processing; however, they lack interpretability. Radiomics, on the other hand, requires time-consuming, (semi-)automatic image segmentation, but allows for some interpretability of the model [4, 5, 8]. In our study, 2 radiomics features ranked in the top 5 among all variables: first, original surface volume ratio (SA:V) of tumor in tomosynthesis. The higher the SA:V, the more likely to have residual cancer after NAST. This may indicate that patients with more compact (sphere-like) shaped tumors on tomosynthesis might have higher chances of reaching pCR (e.g., triple-negative tumors) [45], while patients with irregular-shaped, crab-like, and polygonal tumors have lower chances to reach pCR (e.g., luminal tumors). Second, original GLSZM zone entropy of tumor in ultrasound images. GLSZM zone entropy measures the uncertainty/randomness in the distribution of zone sizes and gray levels. A higher value indicates more heterogeneity in the texture patterns [19]. This may indicate that breast tumors with heterogeneous echo on ultrasound images have lower chances to reach pCR.

This study has limitations. First, this is a retrospective, single-center study. Potential selection bias might have affected our findings, as a relevant number of patients who did not undergo imaging at our institution were excluded. Another source of bias arises from the unitary ethnographic information, since, e.g., Asian women tend to have denser breasts [46], which might have a negative influence on the model’s generalizability [47, 48]. Second, our findings will have to be replicated on images taken on different ultrasound and tomosynthesis machines to ensure generalizability of the algorithms. A prospective, multicenter study is required to further validate our findings. Third, tomosynthesis allows for digital reconstruction in 2 planes but not for 3D reconstruction. Thus, a single slice of tomosynthesis planes was analyzed in this study. Future research may look into automatically analyzing video clips of tomosynthesis to capture the full potential of tomosynthesis. Fourth, our analysis spans over a large timeframe from 2010 to 2020, patients underwent a variety of NAST, and the standard of care has changed during these times. As our study focuses on pre-treatment ultrasound images, we do not expect that the response of different NAST on imaging influences our models but we acknowledge that response to NAST has much improved with the use of modern NAST regimens [32, 49]. Thus, the issue of changing in- and output parameter over time might be a point of attention for further research, also when implementing such models in the future in clinical practice. Sixth, different definitions for pCR exist (ypT0, ypN0 vs. ypTis, ypN0). While most guidelines allow residual in situ disease to be considered a complete response, our present study was performed with an eye to potentially exclude residual cancer early to reduce surgical management. Thus, also, in situ disease must be excluded (indication for surgical resection) which is why we chose this endpoint, in line with previous research on this topic [35]. We provided a comparison of the integrative multi-modal model’s performance on different definition of pCR (ypT0, ypN0 vs. ypT0/is, ypN0) in Table S6. Seventh, ultrasound presents an inherent inter-rater variability which also applies to radiomics-based ultrasound analysis. Thus, future studies are needed to confirm reproducibility of the features. In order to minimize feature bias during the radiomics analysis, we used fixed bin widths for image discretization and outlier removal techniques for re-segmentation, which complies with recent guidelines and other research in that area [15, 50].

## Conclusion

We developed and compared intelligent algorithms using multi-modal pretreatment ultrasound and tomosynthesis radiomics features in addition to clinical variables to predict response to NAST in breast cancer prior to treatment initiation. The integrative, multi-modal algorithm showed significant improvement in assessing response to NAST compared to an algorithm using clinical variables only (AUC 0.81, 95% CI 0.75–0.87 vs. AUC 0.72, 95% CI 0.65–0.78, *p* = 0.007) with a FNR of 6.7% in the validation set (10 of 150 patients with missed residual cancer in the surgical specimen, ypT+ or ypN+). The FNR of the multi-modal pretreatment ultrasound and tomosynthesis radiomics model was in range with previous yet more invasive efforts of reliably excluding residual cancer after NAST using minimally invasive biopsies. Further prospective validation of our findings seems warranted to confirm our results and enable individualized predictions of NAST outcomes prior to treatment initiation.

### Supplementary information

ESM 1 (PDF 257 kb)

## Abbreviations

AUC: Area under the curve
CI: Confidence interval
DCA: Decision curve analysis
DICOM: Digital imaging and communications in medicine
ER: Estrogen receptor
FNR: False-negative rate
FPR: False-positive rate
GLSZM: Gray level size zone matrix
HER-2: Human epidermal growth factor receptor 2
ML: Mediolateral
MLO: Mediolateral oblique
NAST: Neoadjuvant systemic treatment
NPV: Negative-predictive value
PCCM: Pearson correlation coefficient matrix
pCR: Pathologic complete response
PPV: Positive-predictive value
PR: Progesterone receptor
RFE: Recursive feature selection
SVM: Supporting vector machine

## Declarations

### Guarantor

The scientific guarantor is André Pfob.

### Statistics and Biometry

No complex statistical methods were necessary for this paper.

### Study subjects or cohorts overlap

This study cohort has not been previously reported.

### Methodology

- retrospective
- diagnostic study
- performed at one institution
