# Supplementary material for: Can multi-modal radiomics using pretreatment ultrasound and tomosynthesis predict response to neoadjuvant systemic treatment in breast cancer?
Source: Eur Radiol. 2023 Sep 14;34(4):2560–73. doi: 10.1007/s00330-023-10238-6 (PMC10957593; doi:10.1007/s00330-023-10238-6)
Supplement: Supplementary file 1 — (PDF 257 kb) [file 330_2023_10238_MOESM1_ESM.pdf]

Can multi-modal radiomics using pretreatment ultrasound and tomosynthesis predict response to neoadjuvant systemic treatment in breast cancer?

Electronic Supplementary Material

Hereby provided:

I) a checklist informed by recent guidelines[2] on machine learning in medicine according to Liu et al.[1] (Table S1)

II) a definition of clinical variables used for the algorithm development (Table S2)

III) results of recursive feature elimination for feature selection (Table S3)

IV) all variables used in the integrative multi-modal model and corresponding mean drop out loss (Table S4)

V) different model's AUC value with 95% confidence interval and corresponding hyperparameters in the validation set (Table S5)

VI) AUC values of different models in development set (Figure S1)

VII) calibration plot of the integrative multi-modal model (Figure S2)

VIII) comparison of the integrative multi-modal model performance on different definition of pCR in the validation set. (ypT0N0 vs. ypT0/is, ypN0) (Table S6)

Table S1. A checklist informed by recent guidelines[2] on machine learning in medicine according to Liu et al[1]

Aspect of study		Our study	Considerations
Summary of prediction task		Identify patients with residual cancer (ypT+ and/or ypN+) after neoadjuvant systemic treatment (NAST) prior to the initial of the treatment	What is the clinical utility of having these predictions available?
Data	Input	37: patient, tumor, histopathological features and radiomics features	Are these data easily obtainable in routine clinical workflows?
	Output	2-class: pathological complete response (pCR, ypT0 and ypN0) vs non pCR (ypT+ and/or ypN+)	Is the prediction clinically relevant? Is this prediction at the right granularity, such as a meaningful number of patient risk categories?
	Label	pCR was assumed if no residual invasive or in-situ tumor cells were found in the breast and axillary lymph nodes (ypT0 and ypN0), we considered residual disease as ypT+ and/or ypN+ in this study.	Is this an accepted grading scale and is the reference standard reliable?
	Patient population	We included a cohort of 720 patients diagnosed with breast cancer from 2010 to 2020 who underwent neoadjuvant treatment at Heidelberg University Hospital. We randomly split the whole cohort into a development set and a	What are the patient population and inclusion/exclusion criteria? What are the numbers of patients with each label and other characteristics?

		validation set (type 2a study according to TRIPOD guidelines[2]).	
	Development / Validation split	Data from development set were used for the algorithm training and internal testing. 10-fold cross validation was used for the hyperparameter tuning.<br>Model performance were evaluated by validation set.	Do any patients appear in both development and validation sets? Is this a strong study design with respect to evaluation of generalization?
	Amount of data in the development set	504 patients (328 events) in the training and internal testing data	Is this sufficient to develop the ML model, given its complexity?
	Amount of data in the validation set	216 patients (150 events) in the validation set	Is this sufficient to have confidence in the generalizability of the results and any clinically important subgroups?
Machine Learning (ML)	Method	Supporting vector machine (SVM)	Is this a standard or customized method? What are the parameters and how many are there?
	Training process	Random initialization, single 2-class prediction	Was transfer learning (eg, preinitialization), or multi-task learning (eg, multiple predictions) used to help training?
	Data augmentation	Not applicable	Data augmentation typically helps performance and generalization. Was this done and appropriate for this data type?
	Hyperparameters that were optimized	SVM:<br>Gamma:a small	What were the hyperparameter s?

		gamma means a Gaussian with a large variance; large gamma leads to high bias and low variance models, and vice-versa.<br>C: parameter of the soft margin cost function, which controls the influence of each individual support vector	
	Use of tuning set	Yes, 10-fold cross validation	Was a separate tuning set (independent of the final validation set) used for hyperparameter tuning?
	Time taken to apply model per data point	Not reported	Is this amount of time feasible in the context of routine clinical workflows?
Evaluation	Dataset inclusion/exclusion criteria	Please find in the original article Methods$Patients selection	Were any data excluded based on ML predictions? If so, why and does this skew the results?
	Performance metric	Performance of models was mainly evaluated using false negative rate (FNR). Additional diagnostic metrics like area under the receiving operator curve (AUROC), accuracy, specificity, sensitivity, false positive rate (FPR), positive-predictive value (PPV) and negative-predicative value (NPV) were also calculated.Confidence intervals for AUROC	Is this standard? What is “random” performance (eg, 0 or 0.5), and what is perfect (eg, 1)? Is this appropriate given the incidence or prevalence of the predicted label? How were the operating points selected?

		were computed with 2000 stratified bootstrap replicates). The operating point was selected as maximum threshold for which the upper 95% CI of the false-positive rate was <5%. Calibration: calibration plots (observed vs. predicted probability) and Spiegelhalter's Z statistic[3,4]	
	Was this an independent validation set?	Yes, independent validation set	Was this final validation set used to make any ML model development decisions? Including but not limited to hyperparameter tuning, neural network "checkpoint" selection, method selection, etc
	Human comparison metric	Not applicable Performance was compared to previous attempts to Identify patients with residual cancer (ypT+ and/or ypN+) after neoadjuvant systemic treatment (NAST) prior to the initial of the treatment.	Is this a fair comparison: eg, does the human grader have sufficient training (eg, years post residency), information (eg, other clinical variables) and time (eg, comparable with routine practice)?[1] Are the statistics (confidence intervals etc.) present and appropriate?
	Human comparator	Not applicable	Were there any deviations from standard practice? eg, were the human graders provided sufficient time and with

			the full-resolution image?
	Human vs ML model performance	Not applicable	Based on prior literature, are the human performance sensible? Do they over- or under-represent human performance?
	Performance gap in development-validation	No performance gap	What was the gap between tuning and validation, and does this suggest good generalizability?
	Subgroup / sensitivity analysis	No potential confounding factors to report	Are there any potential confounding factors that should be examined more closely (eg, image capturing device manufacturer or model, etc.)?

Table S2. Definition of clinical Variables used for Algorithm Development

Variable	Variable type	Definition
age	numerical (years)	numerical age of patient
menopause status	binary,<br>(1) pre-menopausal<br>(2) peri-menopausal<br>(3) post-menopausal	phases of menopause.
largest diameter on ultrasound before NAST	numerical (mm)	largest diameter showed on pretreatment ultrasound images, evaluated by experienced radiologists
largest diameter on tom-osynthesis before NAST	numerical (mm)	largest diameter showed on pretreatment tomosynthesis images, evaluated by experienced radiologists
tumor type	factor, either<br>(1) Invasive<br>(2) In situ	according to ASCO/CAP and German S3 guidelines, evaluated by board certified pathologist on the initial diagnostic biopsy (not on VAB or surgery specimen)
tumor grading	factor, either<br>(1) G1<br>(2) G2<br>(3) G3<br>(4) G4	according to Elston and Ellis, evaluated by board certified pathologist on the initial diagnostic biopsy (not on VAB or surgery specimen)
estrogen receptor status	binary, positive / negative	according to ASCO/CAP and German S3 guidelines, evaluated by board certified pathologist on the initial diagnostic biopsy (not on VAB or surgery specimen)
progesterone receptor status	binary, positive / negative	according to ASCO/CAP and German S3 guidelines, evaluated by board certified pathologist on the initial diagnostic biopsy (not on VAB or surgery specimen)
Her2Neu receptor status	binary, positive / negative	according to ASCO/CAP and German S3 guidelines, positive if status ≥3 or amplified by FISH/CISH, evaluated by board certified pathologist on the initial diagnostic biopsy (not on VAB or surgery specimen)

Ki-67	numeric	according to ASCO/CAP and German S3 guidelines, evaluated by board certified pathologist on the initial diagnostic biopsy (not on VAB or surgery specimen)
cT status	factor, either<br>(1) T1<br>(2) T2<br>(3) T3<br>(4) T4<br>(5) Tx	local involvement of tumor in breast assessed by either ultrasound and mammography and/or MRI as applicable in clinical routine
cN status	factor, either<br>(1) cN0<br>(2) cN1<br>(3) cN2<br>(4) cN3<br>(5) cNx	axillary lymph node status as assessed by either ultrasound and mammography and/or MRI as applicable in clinical routine
breast density	factor, either<br>(1) fatty<br>(2) scattered fibroglandular tissues<br>(3) heterogenous dense<br>(4) extremely dense	according to BI-RADS system, breasts contain glandular tissue, fibrous connective tissue, and fatty breast tissue, breast density is a term that describes the relative amount of these different types of breast tissue as seen on a mammogram.
outcome variable: residual cancer (ypT+ and/or ypN+)	binary, yes/no	whether residual invasive or in-situ tumor cells were found in the breast and axillary lymph nodes (ypT+ and/or ypN+).

Table S3. Results of recursive feature elimination (RFE) for feature selection

Table S3-1 RFE for first view of ultrasound tumor features

Variables	Accuracy	Kappa	AccuracySD	KappaSD
1	0.5677	0.026262	0.05608	0.12196
2	0.5773	0.014733	0.06956	0.11786
3	0.552	-0.01053	0.09468	0.09691
4	0.5417	-0.00302	0.1169	0.09576
5	0.5567	0.00163	0.11727	0.10368
6	0.5483	0.006744	0.11868	0.10783
7	0.5493	-0.0286	0.1023	0.11903
8	0.5563	-0.00168	0.10674	0.0865
9	0.5537	0.008548	0.11016	0.10904
10	0.546	-0.00632	0.10635	0.12158
11	0.55	-0.01351	0.10118	0.11674
12	0.5633	0.002178	0.09677	0.12342
13	0.5557	-0.01043	0.09058	0.11097
14	0.552	-0.00424	0.09534	0.11399
15	0.573	0.002969	0.07766	0.12166
16	0.5553	-0.00327	0.09298	0.12481
17	0.5507	-0.01373	0.07315	0.10661
18	0.5527	-0.01096	0.07949	0.1291
19	0.5623	-0.0036	0.08031	0.13712
20	0.5613	0.003582	0.0673	0.11421
21	0.5713	0.00896	0.07031	0.12046
22	0.5753	0.012631	0.07057	0.13674

Top 2 variables: HToriginal_glszm_ZoneVariance, HToriginal_glszm_LargeAreaEmphasis

Table S3-2 RFE for first view of ultrasound peritumor features

Variables	Accuracy	Kappa	AccuracySD	KappaSD
1	0.565	2.54E-03	0.07797	0.11551
2	0.5663	-1.86E-02	0.06611	0.12071
3	0.574	1.79E-03	0.07348	0.12361
4	0.5707	-1.63E-02	0.07123	0.11749
5	0.558	-2.80E-02	0.0816	0.12517
6	0.5727	-2.15E-05	0.0808	0.09958
7	0.5673	-8.14E-03	0.08282	0.0966
8	0.5677	9.84E-04	0.0986	0.09333
9	0.543	-1.08E-02	0.10164	0.09627
10	0.5443	-1.23E-02	0.09341	0.09945
11	0.5413	-6.83E-03	0.08304	0.11065
12	0.5397	-3.65E-03	0.08663	0.11857
13	0.5467	-7.86E-03	0.08864	0.11662
14	0.5487	2.64E-03	0.08721	0.12265
15	0.5487	1.35E-02	0.09421	0.1192
16	0.516	1.16E-03	0.10701	0.12088
17	0.5307	8.57E-03	0.10808	0.1123
18	0.514	1.21E-02	0.10645	0.1033
19	0.5253	2.97E-02	0.11558	0.10968
20	0.492	5.66E-03	0.09561	0.09596
21	0.496	3.24E-02	0.10071	0.13269
22	0.4973	3.61E-02	0.09755	0.12185

Top 3 variables of first view ultrasound features: HMoriginal_shape_SurfaceVolumeRatio, HMdiagnostics_Image-original_Mean, HMoriginal_firstorder_Minimum.

Table S3-3 RFE for second view of ultrasound tumor features

Variables	Accuracy	Kappa	AccuracySD	KappaSD
1	0.5270	-0.01437	0.07513	0.1411
2	0.6023	0.03357	0.05665	0.1284
3	0.6453	0.07391	0.05605	0.1299
4	0.6180	0.07800	0.06716	0.1305
5	0.6253	0.11090	0.08201	0.1332
6	0.6293	0.12856	0.07690	0.1221
7	0.6193	0.12322	0.08353	0.1260
8	0.6323	0.15170	0.06294	0.1172
9	0.6153	0.12862	0.06741	0.1252
10	0.6157	0.13283	0.07256	0.1352
11	0.6157	0.13408	0.06904	0.1119
12	0.6230	0.14597	0.07663	0.1384
13	0.6390	0.17084	0.07156	0.1256
14	0.6323	0.14920	0.06986	0.1359
15	0.6373	0.15908	0.06525	0.1227
16	0.6257	0.13945	0.07278	0.1324
17	0.6367	0.16983	0.06768	0.1439
18	0.6327	0.15464	0.07911	0.1442
19	0.6440	0.16417	0.06288	0.1416
20	0.6427	0.15660	0.06861	0.1450
21	0.6317	0.14559	0.07121	0.1275
22	0.6290	0.15230	0.07662	0.1393

Top 3 variables: VTdiagnostics_Mask-original_VoxelNum, VToriginal_glszm_ZoneEntropy, VToriginal_ngtdm_Coarseness.

Table S3-4 RFE for second view of ultrasound peritumor features

Variables	Accuracy	Kappa	AccuracySD	KappaSD
1	0.5423	0.00578	0.07448	0.14435
2	0.5857	0.0437	0.06344	0.09301
3	0.5603	0.05002	0.09653	0.1143
4	0.554	0.06551	0.1083	0.11397
5	0.5753	0.07759	0.08366	0.11684
6	0.6007	0.05918	0.06589	0.10906
7	0.6347	0.09094	0.05984	0.11652
8	0.6577	0.11274	0.04495	0.1136
9	0.657	0.10735	0.05119	0.13141
10	0.6693	0.12681	0.04609	0.11474
11	0.662	0.09079	0.04179	0.1106
12	0.6717	0.11441	0.03438	0.09951
13	0.6687	0.09176	0.03542	0.09084
14	0.6663	0.09897	0.03375	0.086
15	0.6633	0.07859	0.03611	0.09736
16	0.661	0.07887	0.02789	0.08569
17	0.6673	0.08471	0.03383	0.10004
18	0.6667	0.08929	0.03658	0.09266
19	0.6667	0.092	0.03282	0.09426
20	0.6657	0.07905	0.0334	0.09637
21	0.671	0.09228	0.03608	0.1053
22	0.671	0.077	0.03008	0.08872

Top 5 variables: VMdiagnostics_Mask-original_VoxelNum, VMoriginal_shape_SurfaceVolumeRatio, VMoriginal_shape_Elongation, VMoriginal_glcm_Correlation, VMoriginal_ngtdm_Coarseness

Table S3-5 RFE for tomosynthesis tumor features

Variables	Accuracy	Kappa	AccuracySD	KappaSD
1	0.4835	-0.01612	0.0669	0.11143
2	0.577	0.07284	0.12501	0.16255
3	0.6137	0.05013	0.10678	0.15314
4	0.6429	0.11497	0.073	0.13762
5	0.6246	0.07249	0.06235	0.13546
6	0.6114	0.03011	0.06641	0.1325
7	0.6469	0.02366	0.0579	0.07472
8	0.6427	0.05	0.05171	0.11265
9	0.6461	0.08063	0.05383	0.12518
10	0.636	0.05804	0.04554	0.11068
11	0.632	0.06507	0.05332	0.09537
12	0.6278	0.035	0.05989	0.10057
13	0.6114	0.02327	0.06648	0.08439
14	0.6365	0.05768	0.04937	0.08131
15	0.6343	0.03969	0.04096	0.1229
16	0.6357	0.06734	0.0555	0.12142
17	0.6437	0.06757	0.04359	0.12088
18	0.6368	0.03983	0.04833	0.0889
19	0.6301	0.06175	0.06458	0.12608
20	0.6258	0.05078	0.05417	0.10594
21	0.6233	0.02872	0.05582	0.10021
22	0.6371	0.03583	0.05256	0.11553
23	0.6257	0.02656	0.06858	0.09665
24	0.6323	0.02937	0.05382	0.10183
25	0.6275	0.02783	0.06181	0.09074
26	0.6342	0.01529	0.05491	0.11692
27	0.6296	0.04665	0.06025	0.10856
28	0.635	0.03519	0.04915	0.09868
29	0.6267	0.03464	0.04947	0.09912
30	0.6305	0.04168	0.05692	0.09595
31	0.6306	0.02624	0.06462	0.10036
32	0.6284	0.05765	0.06538	0.1191
33	0.6235	0.02653	0.05762	0.08389

Top 5 variables: TToriginal_shape_SurfaceVolumeRatio, TToriginal_shape_Sphericity, TToriginal_shape_Maximum3DDiameter, TToriginal_shape_Maximum2DDiameterSlice, TToriginal_shape_Maximum2DDiameterColumn.

Table S3-6 RFE for tomosynthesis peritumor features

Variables	Accuracy	Kappa	AccuracySD	KappaSD
1	0.6167	7.55E-02	0.05855	0.138918
2	0.6597	4.04E-02	0.026488	0.072481
3	0.661	-2.78E-03	0.01286	0.026179
4	0.65	-5.11E-03	0.024176	0.061821
5	0.6531	-6.23E-03	0.02735	0.066915
6	0.6583	4.14E-03	0.023896	0.056217
7	0.6582	-4.14E-03	0.018381	0.039477
8	0.6631	1.45E-02	0.016246	0.059463
9	0.6621	6.30E-03	0.011476	0.031286
10	0.6624	7.65E-03	0.013178	0.054665
11	0.6645	8.99E-03	0.011073	0.039955
12	0.6635	2.02E-03	0.006495	0.015756
13	0.6635	2.73E-03	0.009058	0.022346
14	0.6628	6.43E-04	0.009685	0.019907
15	0.6631	1.36E-03	0.00614	0.006547
16	0.6635	3.93E-03	0.00859	0.021551
17	0.6614	-4.64E-03	0.01032	0.016223
18	0.6607	-4.76E-03	0.010463	0.020351
19	0.6621	-1.53E-03	0.007437	0.01718
20	0.6604	-5.43E-03	0.010094	0.016872
21	0.6635	-6.59E-05	0.006481	0.011578
22	0.6635	-1.17E-05	0.006169	0.006908
23	0.6628	-5.45E-05	0.006726	0.015173
24	0.6631	-1.87E-05	0.007045	0.013655
25	0.6628	-2.05E-03	0.007017	0.008181
26	0.6611	-3.94E-03	0.01121	0.019702
27	0.6624	-2.71E-03	0.008603	0.01342
28	0.6642	2.67E-03	0.007677	0.017898
29	0.6614	-4.71E-03	0.009506	0.01659
30	0.6642	2.09E-03	0.007368	0.014651
31	0.6642	1.92E-03	0.006749	0.013793
32	0.6631	-4.21E-05	0.006425	0.009468
33	0.6628	-2.02E-03	0.006379	0.008085

Top 5 variables: TMoriginal_ngtdm_Coarseness, TMoriginal_shape_Sphericity, TMoriginal_glrlm_RunVariance, TMdiagnostics_Mask-original_VolumeNum, TMoriginal_ngtdm_Strength.

Table S4. All variables used in the integrative multi-modal model and corresponding mean drop out loss.

Variable name	mean dropout loss
Clinical variables	
Age	0.5579
Menupause status	0.5582
Largest diameter on ultrasound before NAST	0.5589
Largest diameter on tomosynthesis before NAST	0.5594
Grading	0.5577
Tumor type	0.5587
ER	0.5547
PR	0.5565
HER-2	0.5546
Breast density	0.5583
cT	0.5584
cN	0.5565
Ki67	0.5566
Karnofsky index	0.5595
First view ultrasound features	
tumor GLSZM large area emphasis	0.5575
tumor GLSZM zone variance	0.5587
pertitumor diagnostics image original mean	0.5576
pertitumor first order minimum	0.5576
perittumor shape surface volume ratio	0.5584
Second view ultrasound features	
tumor GLSZM zone entropy	0.5556
tumor NGTDM coarseness	0.5577

tumor diagnostics mask original voxel num	0.5583
peritumor shape surface volume ratio	0.5571
peritumor shape elongation	0.5579
peritumor GLCM correlation	0.5582
peritumor NGTDM coarseness	0.5582
peritumor diagnostics mask original voxel num	0.5582
Tomosynthesis features	
tumor shape surface volume ratio	0.5482
tumor shape maximum 3D diameter	0.5586
tumor shape maximum 2D slice	0.5586
tumor shape sphericity	0.5588
tumor shape maximum 2D diameter column	0.5591
peritumor GLRLM run variance	0.5574
peritumor shape sphericity	0.5583
peritumor diagnostics mask original voxel num	0.5585
peritumor NGTDM coarseness	0.5586
peritumor NGTDM strength	0.5589

Table S5. Different model's AUC value with 95% confidence interval and corresponding hyperparameters

Model	AUC	95%CI	degree	scale	C
only clinical	71.8	65.1-78.2	1	0.000635	127
clinical + one US tumor	72.8	65.5-78.0	2	0.000001	0.6002
clinical + one US tumor and peritumor	73.2	66.7-79.4	2	0.054200	0.0695
clinical + double US tumor	74.8	70.1-82.5	1	0.000001	15.6
clinical + double US tumor and peritumor	76.9	70.4-82.8	2	0.000001	0.6
clinical + tomo tumor	77.6	70.7-84.1	1	0.000774	16.7
clinical + tomo tumor and peritumor	78.6	71.5-85.1	2	0.054200	0.0695
final model	81.0	74.6-86.9	1	0.000774	16.7

abbreviations: US, ultrasound; tomo, tomosynthesis.

Table S6. Comparison of the integrative multi-modal model performance on different definitions of pCR in the validation set. (ypT0N0 vs. ypT0/is, ypN0)

Validation set (n=216)		Pathologically confirmed		AUC (95%CI)
		non pCR	pCR	
Integrative multi-modal model (pCR: ypT0 and ypN0)	residual cancer	140	41	0.81 (0.75-0.87)
	no residual cancer	10	25	
Integrative multi-modal model (pCR: ypT0/is and ypN0)	residual cancer	128	84	0.78 (0.71-0.85)
	no residual cancer	0	4	

Figure S1. Different models' AUC value with 95% confidence interval in the development set

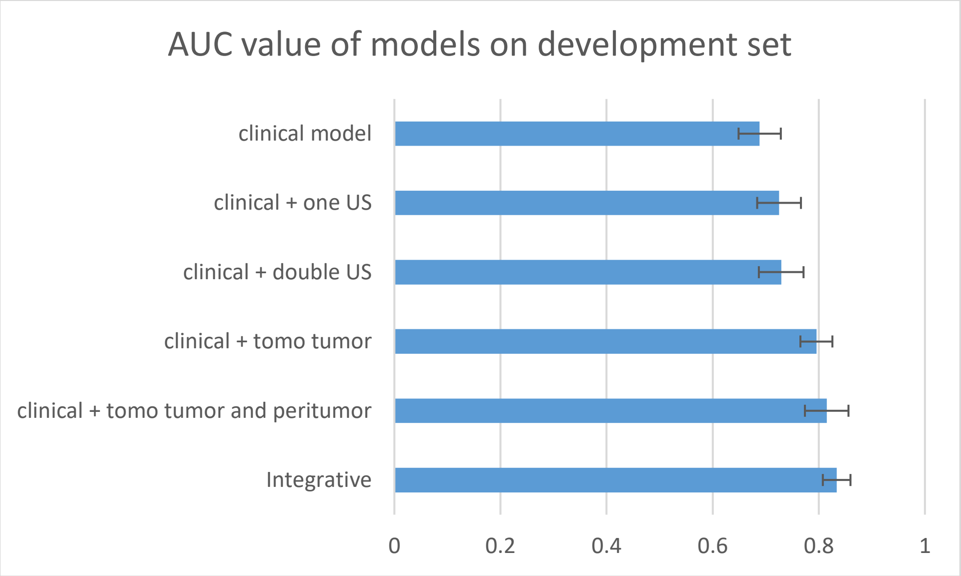

Abbreviation: AUC, area under the curve; US, ultrasound; tomo, tomosynthesis.

Figure S2. Calibration plot of the integrative multi-modal model

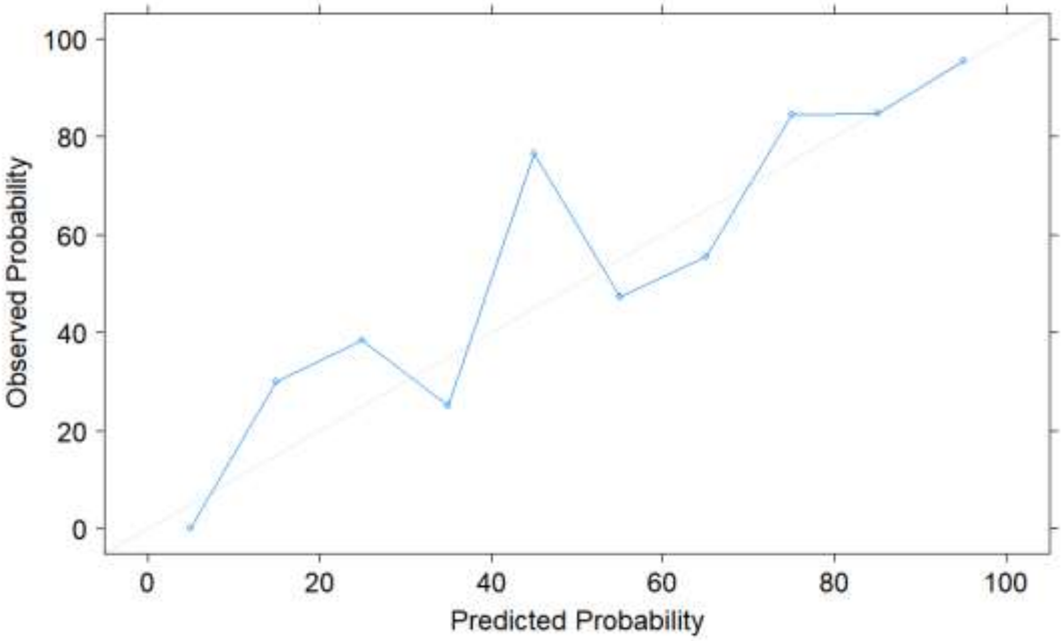

References:

1. Liu Y, Chen P-HC, Krause J et al (2019) How to Read Articles That Use Machine Learning: Users' Guides to the Medical Literature. JAMA 322:1806–1816.

2. Collins GS, Reitsma JB, Altman DG et al (2015) Transparent Reporting of a multivariable prediction model for Individual Prognosis or Diagnosis (TRIPOD): the TRIPOD statement. Ann Intern Med 162:55–63.

3. Harrell FE, Lee KL, Mark DB (1996) Multivariable prognostic models: issues in developing models, evaluating assumptions and adequacy, and measuring and reducing errors. Stat Med 15:361–387.

4. Spiegelhalter DJ (1986) Probabilistic prediction in patient management and clinical trials. Stat Med 5:421–433.
